# Differentiating Alcohol and Substance Use Disorders Using Multiclass Machine Learning Models Based on Routine Hemogram Parameters

**DOI:** 10.3390/healthcare14070904

**Published:** 2026-03-31

**Authors:** Azad Asaf, Yavuz Selim Ogur, Ayşe Erdoğan Kaya, Oğuz Kızılaslan, Münevver Tunçkal, Beyza Erdoğan Aktürk, Ece Yazla

**Affiliations:** 1 Department of Child and Adolescent Psychiatry, Hitit University Çorum Erol Olçok Education and Research Hospital, Çorum 19040, Türkiye; dr.azadasafov@gmail.com; 2 Department of Psychiatry, Sakarya University, Sakarya 54290, Türkiye; 3 Department of Psychiatry, Hitit University Çorum Erol Olçok Education and Research Hospital, Çorum 19040, Türkiye; dr.ayserdogan@gmail.com (A.E.K.); oguzredlion@gmail.com (O.K.); dr.mtunckal@gmail.com (M.T.); eceyazla@yahoo.com (E.Y.); 4 Department of Psychiatry, Tarsus State Hospital, Mersin 33460, Türkiye; beyzaerdogan128@gmail.com

**Keywords:** substance dependence, alcohol dependence, hematological parameters, biomarker, machine learning

## Abstract

**Highlights:**

**What are the main findings?**

- Routine hemogram parameters combined with multiclass machine learning models successfully differentiated alcohol use disorder, substance use disorder, and healthy controls.
- The Random Forest algorithm demonstrated the highest diagnostic performance, achieving an accuracy of 81.6% and an AUC of 0.93.

**What are the implications of the main findings?**

- Low-cost, widely available hematological parameters may serve as practical peripheral biomarkers in addiction medicine.
- Machine learning-based analysis of routine laboratory data may support clinical decision-making and screening in psychiatric practice.

**Abstract:**

**Aim:** The identification of alcohol and substance use disorders is primarily based on clinical evaluation, and the lack of accessible objective markers remains a challenge. This study aimed to explore whether routine hemogram parameters, analyzed using multiclass machine learning models, could assist in differentiating individuals with alcohol use disorder, substance use disorder, and healthy controls. **Method:** This retrospective case–control study included 35 patients with alcohol use disorder, 61 patients with substance use disorder, and 132 healthy controls. Routine hematological parameters were obtained from hospital records. Multiclass classification models, including Random Forest, Support Vector Machine (SVM), Artificial Neural Network (ANN), and other conventional machine learning algorithms, were applied. Model performance was evaluated using 10-fold cross-validation with metrics including accuracy, sensitivity, precision, F1-score, and AUC. **Results:** Significant differences were observed among groups in several hematological parameters, including monocyte count, basophil count, and RDW-CV (*p* < 0.05). Among the models, Random Forest achieved the highest overall accuracy (81.6%) and AUC (0.93), followed by SVM and ANN models with comparable performance. However, classification performance was not uniform across all classes, and sensitivity was relatively lower for the alcohol use disorder group compared to the control and substance use disorder groups. **Conclusions:** These findings suggest that machine learning models based on routine hemogram parameters may provide a preliminary, low-cost, and accessible supportive approach for differentiating addiction-related conditions. However, the results should be interpreted as exploratory, given the retrospective single-center design, limited sample size, lack of external validation, and absence of model interpretability analyses. Further studies incorporating larger multicenter datasets, confounding factors, and explainable artificial intelligence methods are required before clinical application can be considered.

## 1. Introduction

Alcohol and substance use disorders constitute an increasingly prevalent public health problem worldwide, imposing substantial economic, social, and legal burdens on societies [1]. Diagnostic assessment primarily relies on psychiatric evaluations based on the Diagnostic and Statistical Manual of Mental Disorders, Fifth Edition (DSM-5) and the International Classification of Diseases, Tenth Revision (ICD-10) criteria; however, toxicological analyses of biological samples such as blood, urine, and hair are also frequently employed [2,3]. Nevertheless, the expanding diversity of addictive substances, incomplete understanding of addiction pathophysiology, limited transparency in patient self-reporting, and the absence of validated diagnostic biomarkers continue to complicate the diagnostic process. At this stage, peripheral hematological parameters should be considered candidate or potential supportive markers rather than definitive biomarkers of addiction. In addition to clinical challenges, the lack of objective and easily accessible biomarkers may further complicate diagnostic decision-making in forensic psychiatric settings, where accurate identification of substance use disorders carries important legal and judicial implications.

Although biomarker-based approaches substantially support diagnosis and treatment decisions in many non-psychiatric medical disciplines, psychiatry still lacks widely accepted objective tools. Clinical characteristics frequently observed in individuals with addiction—such as impaired risk perception, impulsivity, dopaminergic dysregulation of the brain reward system, heightened reward-seeking behavior, reduced psychological resilience, and adverse social and environmental conditions—highlight addiction as a multifactorial brain disorder [4,5]. Moreover, the genetic, metabolic, and biochemical mechanisms underlying addiction have not yet been fully elucidated.

Given the heterogeneous and complex nature of addiction, multidisciplinary approaches integrating multiple biomarkers appear more consistent with its pathophysiology. However, conventional statistical methods may be insufficient for analyzing multidimensional biomarker data, thereby bringing artificial intelligence (AI) and machine learning (ML) techniques to the forefront [6]. Through data-driven pattern recognition, these methods offer the potential to identify complex disease-related signatures and support clinical decision-making in psychiatry.

Recent studies increasingly suggest that ML-based approaches may be useful in addiction research. A review published in 2023 reported that individuals with substance use disorders could be distinguished from healthy controls with high accuracy using AI-based analyses of neuroimaging data [7]. In a 2020 study from Bangladesh, nine different ML algorithms were applied to predict substance use risk, with logistic regression achieving the highest accuracy (~97.9%), whereas Classification and Regression Trees (CARTs) demonstrated relatively poor performance following principal component analysis [8]. Similarly, a study conducted in Türkiye in 2023 reported that Gaussian SVM achieved the highest prediction accuracy (90.6%) when estimating future substance use risk among individuals already using addictive substances [9].

In addition, substance use disorder represents a clinically heterogeneous condition, encompassing a wide range of substances, patterns of use, and associated psychiatric and medical comorbidities. This heterogeneity may influence both biological parameters and model performance, and should be considered when interpreting findings derived from such datasets.

Studies based on routine laboratory parameters further support the potential of ML in this field. Pinar-Sanchez et al. (2022) demonstrated that a Naive Bayes-based model using more than 60 commonly measured blood parameters achieved approximately 87.5% accuracy in screening for alcohol use disorder [10]. Analyses of nationally representative NHANES data in the United States reported high AUC values for distinguishing moderate drinkers from abstinent individuals using combined biochemical and demographic variables, with improved performance compared to biochemical markers alone [11]. From a prognostic perspective, Raabe et al. (2020/2021) showed that classical biomarkers such as the AST/ALT ratio and mean corpuscular volume (MCV) could contribute to relapse risk stratification following withdrawal treatment [12]. More recently, studies from Türkiye have suggested that novel inflammatory indices, including NLR and MHR, may exhibit moderate discriminative potential, particularly in subgroups with normal liver enzyme levels [13].

In the context of illicit substance use, Chen et al. (2025) developed an explainable LightGBM model based on routine hemogram and biochemical features, reporting high AUC values in both internal and external validation, and identifying routinely measured parameters as key contributors to model performance [14].

Despite these promising findings, the number of studies proposing practical, low-cost, and routinely applicable biomarkers for addiction remains limited, and no consistent peripheral biomarker has yet been established. Accordingly, the present study aimed to explore whether routine peripheral blood–derived hematological parameters, analyzed using multiple machine learning models, may assist in differentiating individuals with alcohol use disorder, substance use disorder, and healthy controls, and to assess the feasibility of developing a cost-effective and clinically scalable diagnostic framework. In this context, the inclusion of both Artificial Neural Networks (ANNs) and conventional machine learning algorithms was intended to evaluate whether different computational approaches provide complementary insights into the modeling of hematological data in alcohol and substance use disorders. Such an approach may be particularly relevant in resource-limited clinical settings, where access to advanced diagnostic tools is limited and scalable, data-driven support systems may offer practical clinical value.

## 2. Materials and Methods

In this study, the potential of routine hemogram parameters to differentiate individuals with alcohol use disorder, substance use disorder, and healthy controls was evaluated using multiclass machine learning approaches. The analytical workflow was structured to include data preprocessing, multiclass labeling, model training, cross-validation, and performance evaluation.

### 2.1. Study Design and Participants

This retrospective case–control study was conducted using a dataset derived from laboratory test results obtained at the time of discharge from patients who were hospitalized at the Alcohol and Substance Use Disorders Treatment Center (AMATEM) of Hitit University Faculty of Medicine between 1 January 2019 and 30 December 2024. The study population consisted of 35 patients diagnosed with alcohol use disorder, 61 patients diagnosed with substance use disorder, and 132 healthy control individuals with no history of psychiatric disorders. Healthy controls were selected from individuals evaluated as part of routine health check-ups.

To minimize the potential confounding effects of demographic variables on classification performance, the groups were constructed to be balanced in terms of age and sex. Ethical approval for the study was obtained from the Hitit University Faculty of Medicine Research Ethics Committee on 12 March 2025 (Decision No: 2025-48). As the study utilized anonymized retrospective data, the requirement for informed consent was waived. All procedures were conducted in accordance with ethical standards and the principles of the Declaration of Helsinki.

### 2.2. Laboratory Parameters

Routine hemogram parameters obtained from peripheral venous blood samples of all participants were analyzed. All measurements were performed using standard automated hematology analyzers (Sysmex XN-1000, Kobe, Japan). All blood samples were obtained at the time of discharge following inpatient treatment. Therefore, the measured hematological parameters may reflect the effects of detoxification, abstinence, hospitalization, treatment, and partial clinical stabilization, rather than baseline biological states associated with the disorder.

The evaluated parameters included white blood cell count, red blood cell count, hemoglobin, hematocrit, mean corpuscular volume, mean corpuscular hemoglobin, mean corpuscular hemoglobin concentration, platelet count, mean platelet volume, platelet distribution width, plateletcrit, and red blood cell distribution width. All laboratory data were retrospectively retrieved from the hospital information management system and transferred to a digital format for analysis.

### 2.3. Data Preprocessing and Statistical Analysis

Prior to the machine learning analyses, the dataset underwent a comprehensive data preprocessing procedure. Records containing missing data were completely excluded from the dataset, and no data imputation or correction procedures were applied. This approach was adopted to prevent the introduction of artificial variance during the modeling process and to ensure that the results were based solely on actual clinical measurements.

Descriptive statistics and group comparisons were performed using SPSS Statistics version 27.0. As the variables did not meet the assumption of normal distribution, non-parametric Kruskal–Wallis H tests were used for intergroup comparisons. A *p*-value < 0.05 was considered statistically significant. Descriptive statistics are presented as the mean ± standard deviation for readability, although non-parametric tests were used due to the non-normal distribution.

The preprocessed dataset was converted Into “.csv” format and subsequently imported into the Python environment for further analysis.

### 2.4. Multiclass Labeling Approach

A multiclass classification approach was applied for the machine learning analyses. Class labels representing dependency status were encoded as follows: 0 = healthy control, 1 = alcohol use disorder, and 2 = substance use disorder. To more clearly evaluate the effect of biological parameters on classification performance, age and sex variables were not included in the models.

### 2.5. Machine Learning Algorithms

During the classification process, Naive Bayes, k-Nearest Neighbors (KNN), Decision Trees, Random Forest, Extreme Gradient Boosting (XGBoost), and Artificial Neural Network (ANN) algorithms were applied. All models were trained and tested on the same dataset, and their performances in differentiating alcohol use disorder, substance use disorder, and healthy control groups were compared. These algorithms were selected due to their widespread use in biomedical classification studies and their demonstrated effectiveness [1].

In particular, the inclusion of both ANN and conventional machine learning algorithms allowed for comparison between neural and traditional classifiers in order to assess which modeling strategies were better suited to the structure of hematological data in addiction research.

Hyperparameter optimization for all models was performed using the GridSearchCV method, and the optimal parameter set for each algorithm was determined. The optimization process was implemented to maximize model performance while preventing overfitting.

The preprocessing and classification steps were implemented within a unified pipeline structure to prevent data leakage during cross-validation. Hyperparameter optimization was performed using GridSearchCV within the cross-validation framework.

In each fold of the 10-fold cross-validation, preprocessing steps were applied exclusively to the training data and subsequently used to transform the test data, ensuring proper separation between training and evaluation processes.

### 2.6. Model Training and Cross-Validation

To enhance the generalizability of the models and reduce the risk of overfitting, 10-fold cross-validation was applied. In each iteration, 90% of the dataset was used for training and 10% for testing, and performance metrics were reported based on the mean values obtained across all folds. The overall workflow consisted of sequential steps including data preprocessing, feature scaling, model training, hyperparameter optimization, and performance evaluation within a cross-validation framework. All performance metrics were averaged across the 10 folds to provide a more stable and reliable estimate of model performance.

### 2.7. Performance Evaluation and Formulas

Model performance was assessed using a 3 × 3 confusion matrix, from which accuracy, sensitivity, precision, and F1-score were calculated using the formulas provided below. Additionally, receiver operating characteristic (ROC) curves were generated for each model, and the corresponding area under the curve (AUC) values were obtained. Particular emphasis was placed on sensitivity, precision, F1-score, and AUC, as reliance on accuracy alone may be insufficient in alcohol and substance use disorders, especially in the presence of class imbalance and clinically relevant false-negative classifications.

$$\mathrm{Accuracy}\;=\;\frac{{\mathrm{TP}}_{1}\;+\;{\mathrm{TP}}_{2}\;+\;{\mathrm{TP}}_{3}}{N}\;\times \;100$$

$${\mathrm{Precision}}_{i}=\frac{{\mathrm{TP}}_{i}}{{\mathrm{TP}}_{i}\;+\;{\mathrm{FP}}_{i}}$$

$${\mathrm{Sensivity}}_{i}\;=\;\frac{{\mathrm{TP}}_{i}}{{\mathrm{TP}}_{i}\;+\;{\mathrm{FN}}_{i}}$$

$${F1\;-\;\mathrm{score}}_{i}\;=\;2\;\times \;\frac{{\mathrm{Precision}}_{i}\;\times \;{\mathrm{Sensitivity}}_{i}}{{\mathrm{Precision}}_{i}\;+\;{\mathrm{Sensitivity}}_{i}}$$

### 2.8. Hyperparameter Optimization

To maximize model performance, hyperparameter optimization was conducted using the GridSearchCV (Grid Search Cross-Validation) approach. For each algorithm, a predefined range of hyperparameters was systematically explored using 10-fold cross-validation. GridSearchCV is an exhaustive hyperparameter tuning method that evaluates all possible combinations within a specified parameter grid, coupled with cross-validation to assess model performance for each configuration. In this procedure, the model is trained and validated for every joint specification of hyperparameters (i.e., the Cartesian product of candidate parameter values), and the optimal parameter set is selected based on the best average validation score across the folds. The “CV” component of GridSearchCV refers to the use of k-fold cross-validation on the training data, enabling a robust estimation of generalization performance while tuning the model. All optimization procedures were implemented using the Python scikit-learn library (version 1.3.0) [15,16].

### 2.9. ROC Curves and AUC Analysis

To assess the discriminative power of the classification models, receiver operating characteristic (ROC) curves were generated and area under the curve (AUC) values were calculated. Due to the multiclass structure of the problem, ROC–AUC evaluations were interpreted using a one-vs-rest approach, in which each class was evaluated against all other classes.

## 3. Results

When the healthy control group (n = 132), alcohol use disorder group (n = 35), and substance use disorder group (n = 61) were compared, statistically significant differences were observed among the groups in terms of hematological parameters (Table 1).

The monocyte count (MO#) was 51.46 ± 20.41 in the control group, 64.71 ± 20.38 in the alcohol use disorder group, and 50.21 ± 28.90 in the substance use disorder group, demonstrating a significant intergroup difference (*p* = 0.002). Similarly, the eosinophil count (EO#) was 16.53 ± 18.55, 21.57 ± 12.30, and 14.72 ± 11.14 in the control, alcohol, and substance use disorder groups, respectively, with a statistically significant difference observed (*p* = 0.003).

In addition, the basophil count (BA#) differed significantly among the control (2.38 ± 2.55), alcohol use disorder (3.03 ± 1.71), and substance use disorder (5.03 ± 4.27) groups (*p* < 0.001) (Table 1).

When the percentage values of leukocyte subtypes were examined, lymphocyte percentage (LY%), monocyte percentage (MO%), neutrophil percentage (NE%), eosinophil percentage (EO%), and basophil percentage (BA%) differed significantly among the groups (all *p* < 0.01) (Table 1).

The red blood cell count (RBC) was 503.70 ± 86.54 in the control group, 506.94 ± 53.93 in the alcohol use disorder group, and 402.80 ± 196.83 in the substance use disorder group, with a statistically significant difference observed among the groups (*p* = 0.008). The mean corpuscular volume (MCV) values were 836.39 ± 162.88, 924.57 ± 61.56, and 840.97 ± 204.75 in the control, alcohol use disorder, and substance use disorder groups, respectively, demonstrating a significant intergroup difference (*p* < 0.001).

Significant differences were also observed among the groups in terms of mean corpuscular hemoglobin (MCH), mean corpuscular hemoglobin concentration (MCHC), plateletcrit (PCT), platelet distribution width (PDW), and red blood cell distribution width–coefficient of variation (RDW-CV) (*p* < 0.05) (Table 1). In contrast, no statistically significant differences were found among the groups with respect to hemoglobin (HGB), hematocrit (HCT), platelet count (PLT), mean platelet volume (MPV), or white blood cell count (WBC) (*p* > 0.05) (Table 1).

Within the scope of the results, a schematic representation of the classification categories used to evaluate system performance is presented in Table 2, and the parameters and formulas of the performance metrics calculated based on these categories are provided in Table 3.

**Figure 1:**
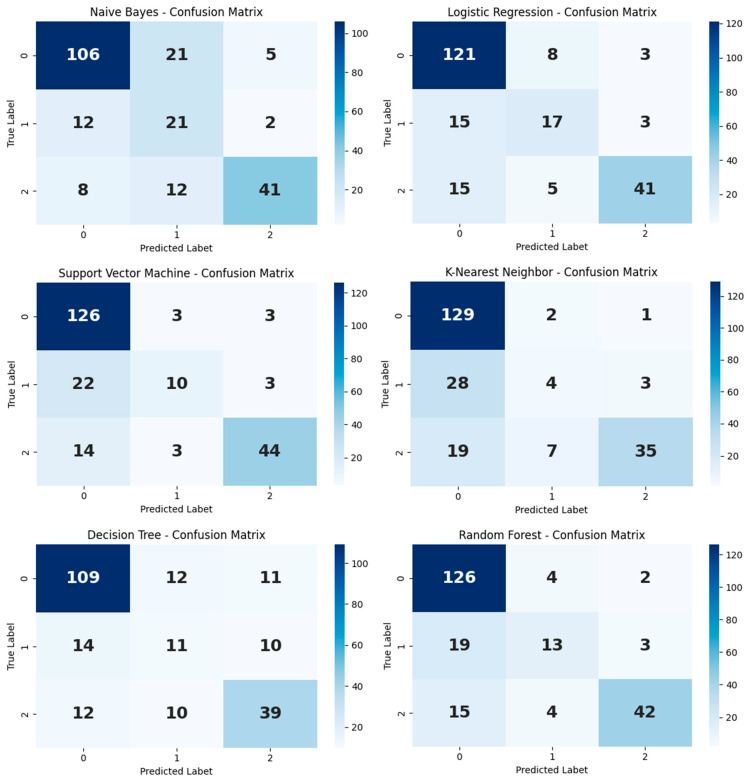

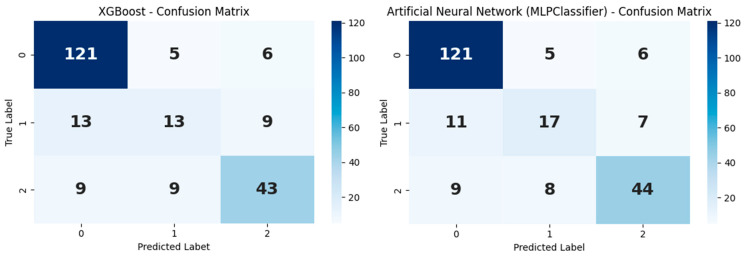
Confusion Matrices.

Examination of the 3 × 3 confusion matrices generated for each model revealed that misclassification rates were lower in the substance use disorder group compared with the other classes, whereas higher interclass confusion was observed in the alcohol use disorder class, likely attributable to its smaller sample size (Figure 1).

As shown in Table 4, the highest classification accuracy was achieved by the Random Forest algorithm (81.6%), followed by Support Vector Machines (80.3%) and Artificial Neural Networks (79.8%). In contrast, Decision Trees and Naive Bayes methods demonstrated relatively lower performance in terms of accuracy and F1-score.

Overall, tree-based models (Random Forest, XGBoost) and neural network-based models produced more balanced results with respect to accuracy and sensitivity. Notably, the discriminative performance of these algorithms was substantially higher in classifying the substance use disorder group (Table 4).

**Figure 2:**
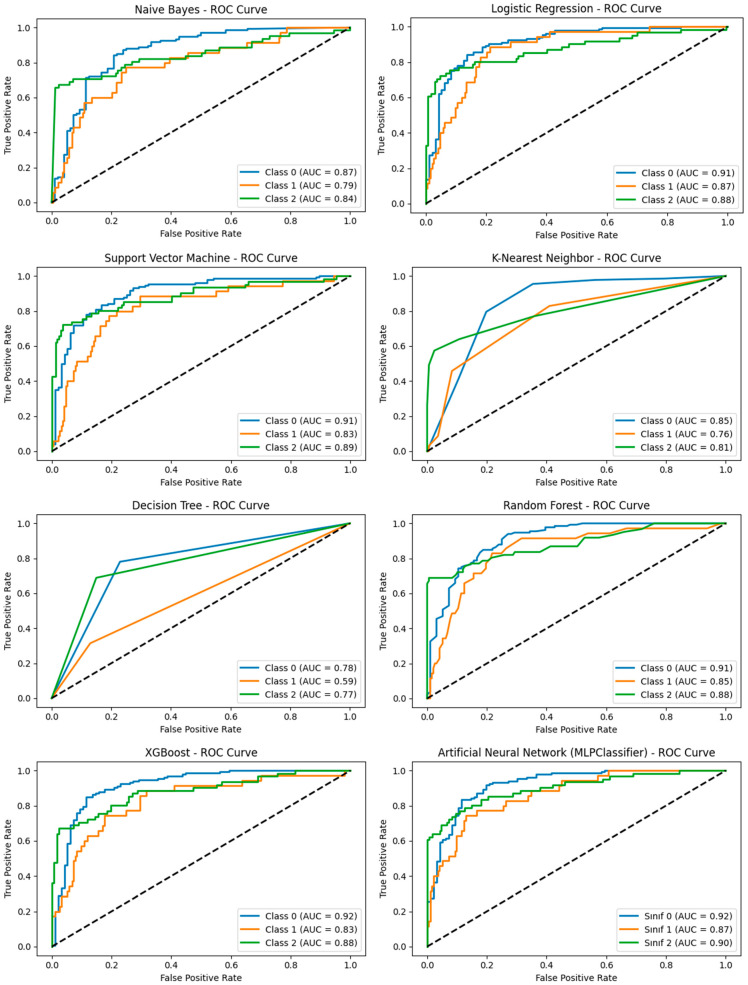
ROC Curves and AUC Values of the Models.

According to the Receiver Operating Characteristic (ROC) analysis, Area Under the Curve (AUC) values across all algorithms ranged from 0.74 to 0.93. The highest AUC value (0.93) was observed for the Random Forest model, indicating its strong discriminative ability among different types of substance dependence. Visual inspection of the ROC curves further demonstrated that the Support Vector Machine (SVM), Artificial Neural Network (ANN), and XGBoost models also exhibited robust performance, with similarly high AUC values (Figure 2).

## 4. Discussion

This study demonstrated that, when analyzed using machine learning algorithms, routine hemogram parameters exhibit high diagnostic potential for distinguishing individuals with alcohol and substance use disorders from healthy controls, achieving an accuracy of 81.6% and an AUC of 0.93. However, these findings should be interpreted as preliminary and exploratory rather than definitive evidence of diagnostic capability. The performance of the Random Forest model in particular suggests that peripheral hematological parameters may serve as candidate supportive markers rather than objective standalone biomarkers in addiction research.

Furthermore, hyperparameter optimization performed using GridSearchCV enhanced the discriminative capability of the models and notably improved the performance of complex algorithms such as Random Forest, Support Vector Machines, and XGBoost when applied to clinical data.

The need for peripheral biomarkers in the field of alcohol and substance use disorders has long been a subject of debate and continues to pose a diagnostic challenge for clinicians [17]. Many studies in the literature have relied on high-cost neuroimaging techniques, such as magnetic resonance imaging (MRI) and functional MRI (fMRI), or on lengthy and comprehensive clinical assessment scales to evaluate brain structure and function in individuals with addiction. However, these approaches present notable limitations in terms of accessibility and scalability in routine clinical practice [18,19,20]. The key contribution of the present study to the existing literature lies in demonstrating that routine hemogram parameters, when analyzed using machine learning approaches, may provide a cost-effective, widely accessible, and easily applicable supportive tool for the identification of alcohol and substance use disorders.

In our study, the significant differences observed in leukocyte subtypes, the neutrophil-to-lymphocyte ratio (NLR), and platelet indices (RDW, PDW, and PCT) between the addiction groups and healthy controls indicate a systematic effect of alcohol and substance use disorders on peripheral hematological parameters. These findings support the notion—consistent with the existing literature—that addiction is a systemic disorder rather than a condition confined solely to the central nervous system, and that it is associated with a chronic inflammatory state [21,22]. However, these hematological alterations should be interpreted with caution, as they may not be specific to addiction-related pathophysiology alone and may also be influenced by nutritional status, inflammatory processes, hepatic dysfunction, medication use, and behavioral factors that were not fully controlled in the present study.

In particular, the increase in the neutrophil-to-lymphocyte ratio (NLR) is widely recognized in the literature as an indicator of chronic low-grade inflammation and oxidative stress [23,24,25]. Activation and increased counts of neutrophils may lead to the release of pro-inflammatory cytokines (e.g., IL-6, TNF-α), whereas the relative reduction in lymphocyte levels may indicate immunosuppression and cell death associated with oxidative damage [13,22,23,26]. This finding is consistent with the growing body of evidence supporting the involvement of both peripheral inflammation and neuroinflammation in the pathophysiology of addiction.

Platelets are cellular components that play an active role in inflammatory processes and can amplify the inflammatory response through the release of cytokines, chemokines, and growth factors, while also contributing to the development of endothelial dysfunction [27]. Alterations in platelet indices (e.g., RDW, PDW) may indicate platelet activation, increased heterogeneity, and disrupted production dynamics associated with alcohol or substance use.

Elevated red cell distribution width (RDW) has been reported in previous studies as a marker of various chronic inflammatory conditions and cardiovascular risk [28,29]. Our findings suggest that this association may also be applicable to the pathophysiology of addiction.

The imbalances observed in leukocyte subtype distributions (e.g., increased monocyte counts or alterations in lymphocyte subsets) in our findings may reflect an adaptive response of the immune system to chronic substance exposure. As also noted in the existing literature, the direct toxic effects of alcohol and certain substances on the bone marrow, as well as their impact on hematopoietic stem cells, may represent another plausible mechanism underlying these hematological alterations [30,31].

Examination of the machine learning model performances indicated that tree-based algorithms (Random Forest, XGBoost), Artificial Neural Networks, and Support Vector Machines outperformed other methods in terms of accuracy and F1-score, whereas Naive Bayes and Decision Tree models demonstrated relatively lower performance. In this study, the superior accuracy and AUC values achieved by the Random Forest algorithm compared with other models may be attributed to its suitability for the structure of biological data. However, although the Random Forest model achieved a high AUC value (0.93), its overall accuracy (81.6%) was relatively lower.

The use of multiple evaluation metrics (accuracy, sensitivity, precision, F1-score, and AUC) enabled a more clinically meaningful interpretation of model performance, as reliance on accuracy alone would have obscured class-specific weaknesses. In particular, the relatively lower sensitivity observed in the alcohol use disorder group suggests that the model may be more effective in distinguishing healthy controls from patient groups than in differentiating between alcohol and substance use disorder subtypes. This limitation is clinically relevant, as reduced sensitivity may increase the risk of under-detection and delayed intervention in alcohol use disorder. Furthermore, this discrepancy may be partly explained by class imbalance, particularly the relatively smaller size of the alcohol use disorder group, which may have affected classification stability and model learning. Taken together, these findings indicate that the discriminatory information is not derived from a single hematological parameter, but rather from a broader multivariable profile involving multiple leukocyte, erythrocyte, and platelet-related features.

The importance of using multiple evaluation metrics in clinical machine learning applications has been emphasized in previous studies, which have shown that reliance on a single metric such as accuracy may lead to misleading conclusions, particularly in imbalanced datasets [32]. In the context of alcohol and substance use disorders, this issue is especially critical, as under-detection of affected individuals may delay diagnosis and treatment. Similarly, prior addiction-related machine learning studies have highlighted the value of comprehensive performance evaluation using metrics such as AUC, sensitivity, and F1-score in improving clinical interpretability [10,33].

The present findings should also be interpreted in light of prior machine learning studies in addiction research. A deep learning study using large-scale Korean survey data reported an AUC of 0.870 for hazardous drinking prediction and found that deep learning outperformed several conventional machine learning methods [33]. Likewise, Pinar-Sanchez et al. reported approximately 87.5% accuracy using a Naive Bayes-based model built on routinely measured blood and medical parameters for alcohol use disorder screening [10]. In contrast, studies from Bangladesh and Türkiye mainly addressed addiction risk prediction rather than multiclass diagnostic discrimination and reported high performance for logistic regression and Gaussian support vector machines, respectively [8,9]. Taken together, these studies suggest that model performance in addiction research is strongly shaped by the prediction target, data modality, and study population. Within this framework, the present study adds preliminary evidence that routine hemogram parameters may offer a low-cost and accessible supportive approach for multiclass classification, while still requiring external validation and better control of confounding factors.

The comparable performance of ANN, SVM, and Random Forest further suggests that hematological data in alcohol and substance use disorders may contain both non-linear and interaction-based patterns that can be captured by different computational approaches. This supports the value of evaluating both neural and conventional machine learning models in clinically heterogeneous datasets.

In addition, trauma-related psychopathology may represent an important contributor to both clinical heterogeneity and biological variability in substance use disorders. Previous studies have shown that trauma exposure is associated with alterations in inflammatory processes, immune regulation, and stress-related neurobiological pathways, which may in turn influence peripheral hematological parameters. Therefore, part of the observed biological variability may be related not only to substance use itself but also to co-occurring trauma-related mechanisms [34,35].

Hemogram parameters consist of biomedical data characterized by non-linear relationships, high feature interactions, and susceptibility to noise. By training a large number of decision trees through random sampling and random feature subspaces, Random Forest reduces the risk of overfitting and enables more effective modeling of complex inter-variable relationships. Compared with single-model approaches, it produces more stable and generalizable predictions and offers a performance advantage in multiclass problems with imbalanced class distributions. These properties may have contributed to the higher discriminative power of the Random Forest algorithm in classifying alcohol use disorder, substance use disorder, and healthy control groups.

The better classification performance observed in the substance use disorder group compared with the other groups suggests that the hematological profile of this group may be more distinctly altered. In contrast, the smaller sample size of the alcohol use disorder group and the presence of potential comorbid medical conditions (e.g., liver disease, nutritional deficiencies) may have contributed to greater heterogeneity in hemogram parameters, thereby limiting the model’s performance for this group.

One of the most important strengths of this study is the attempt to classify addiction status using routine hemogram tests that are already obtained in standard clinical practice. Hemogram testing is a low-cost, rapid, and minimally invasive diagnostic tool available in nearly all healthcare settings. Therefore, machine learning models developed from these parameters may provide clinicians with an additional decision support tool, particularly for screening or risk stratification purposes in addiction medicine.

Such models should not be considered standalone diagnostic tools, but rather complementary approaches to clinical interviews, psychometric assessments, and toxicological analyses. In particular, for patients who are unable or unwilling to provide complete histories, those with limited access to toxicological testing, or those in whom biological response monitoring is desired during follow-up, AI-based models relying on hematological biomarkers may offer objective support to clinical decision-making.

Several additional strengths of the present study merit consideration. First, a three-class classification framework was employed, modeling alcohol use disorder, substance use disorder, and healthy controls as distinct categories rather than collapsing addiction into a single group, thereby better reflecting clinical reality. Second, a comparative evaluation of multiple algorithms—ranging from Naive Bayes to more complex machine learning and neural network–based models—was conducted, allowing for the identification of approaches more suitable for this type of biomedical data. Third, the exclusive use of routine laboratory parameters, which are easily accessible and cost-effective, enhances the clinical applicability of the proposed models. Finally, 10-fold cross-validation was applied to assess model generalizability more reliably than a single train–test split.

Nevertheless, the findings of this study should be interpreted in light of several limitations. First, the study employed a single-center, retrospective design, and the developed models therefore require external validation in independent, multicenter cohorts with diverse patient profiles to ensure their generalizability and robustness across different clinical settings. Second, the unequal distribution of samples across groups, particularly the relatively small size of the alcohol use disorder group, may have reduced statistical power and introduced issues related to class imbalance or overfitting in the machine learning models. Third, although age and sex were balanced across groups, these demographic variables were not included in the classification algorithms; incorporation of such variables in real-world clinical settings may further improve model performance. This modeling choice was made as a deliberate simplification to isolate the discriminative contribution of hematological parameters; however, it reduces clinical realism and should be considered when interpreting the findings.

Fourth, several clinically relevant confounding factors—such as smoking status, nutritional deficiencies, liver dysfunction, comorbid psychiatric disorders, medication use, and inflammatory conditions—were not systematically available due to the retrospective nature of the dataset and therefore could not be included in the analysis. These factors may have influenced both hematological parameters and model performance.

Fifth, the timing of blood sampling at discharge rather than at admission represents an additional limitation, as hemogram values may reflect the effects of detoxification, treatment, hospitalization, and partial clinical stabilization rather than baseline disorder-related biological states. Therefore, the observed hematological alterations may not fully represent baseline pathophysiological mechanisms of addiction and should be interpreted with caution.

Sixth, nested cross-validation was not implemented due to the relatively limited sample size, as such approaches may reduce effective training data and lead to unstable estimates.

Seventh, the present study did not include feature selection procedures or model interpretability analyses (e.g., variable importance ranking or SHAP analysis), which represent important directions for future research to enhance the transparency and clinical interpretability of machine learning-based diagnostic models.

Eighth, the presentation of descriptive statistics as the mean ± standard deviation, despite the use of non-parametric tests, represents a methodological limitation. Although median (interquartile range) values would have been more consistent with the statistical approach, mean ± standard deviation was retained to preserve comparability with the existing literature and the original dataset structure. In addition, some hematological parameters were reported in analyzer-specific scaled formats, which may reduce interpretability and limit comparability with standard clinical units. Due to constraints inherent to retrospective data extraction and standardization, full conversion to conventional clinical units could not be consistently achieved.

Future studies should incorporate detailed clinical and biological confounders—including smoking status, nutritional status, liver function, comorbid conditions, and medication use—to improve model validity and better isolate disorder-specific effects.

Future studies adopting larger, multicenter designs with external validation cohorts will be essential to enhance the generalizability of the developed models.

In addition to routine hemogram parameters, the integration of liver function tests, inflammatory markers (e.g., C-reactive protein [CRP]), and clinical scale scores may enable the development of hybrid models that more comprehensively reflect both the biological and clinical dimensions of addiction.

Moreover, the application of model interpretability techniques—such as feature importance ranking, SHAP, and LIME—may improve clinician trust in these systems and facilitate the identification of clinically meaningful hematological parameters. Finally, longitudinal studies investigating whether such machine learning models can be used not only for diagnostic support, but also for predicting treatment response and relapse risk, would substantially contribute to the development of AI-based clinical decision support systems in the field of addiction medicine.

## 5. Conclusions

In this study, machine learning models based on routine hemogram parameters demonstrated promising ability to differentiate individuals with alcohol and substance use disorders from healthy controls.

However, these findings should be interpreted as preliminary and exploratory in nature. Although the results suggest that routinely available hematological parameters may provide useful supportive information, these models should not be considered standalone diagnostic tools. Further validation in larger, multicenter, and longitudinal studies—along with the inclusion of clinical and biological confounding factors—is required before any potential clinical application can be considered.

Overall, machine learning-based analysis of routine laboratory data may represent a cost-effective and accessible adjunctive approach to support clinical decision-making in addiction medicine.

## Figures and Tables

**Table 1:** Comparison of Hematological Parameters Among Study Groups.

	Control Group (n = 132)	Alcohol Use Disorder (n = 35)	Substance Use Disorder (n = 61)	
MO #	51.4608 ± 20.40628	64.7143 ± 20.38248	50.2131 ± 28.89528	***p* = 0.002**
NE #	415.6866 ± 174.36592	390.0857 ± 196.11272	364.8197 ± 255.46797	*p* = 0.123
EO #	16.5253 ± 18.54800	21.5714 ± 12.29607	14.7213 ± 11.14321	***p* = 0.003**
BA #	2.3779 ± 2.55043	3.0286 ± 1.70614	5.0328 ± 4.26992	***p* < 0.001**
LY%	331.7373 ± 263.82340	368.1714 ± 102.84268	316.5410 ± 118.01590	***p* = 0.005**
MO%	130.1475 ± 176.86084	89.3714 ± 22.84614	311.4754 ± 319.10811	***p* < 0.001**
NE%	593.0184 ± 316.12117	507.3429 ± 116.33343	544.1475 ± 155.68974	***p* < 0.001**
EO%	47.7742 ± 77.80418	30.9143 ± 18.65219	100.4262 ± 119.29843	***p* < 0.001**
BA%	13.4977 ± 33.15286	4.2000 ± 2.48288	47.1639 ± 54.03831	***p* < 0.001**
RBC	503.7005 ± 86.53969	506.9429 ± 53.93075	402.8033 ± 196.82868	***p* = 0.008**
HGB	156.5023 ± 94.81318	158.3714 ± 10.97614	142.5082 ± 37.05699	*p* = 0.059
HCT	443.0876 ± 57.38658	466.3714 ± 35.45924	424.1967 ± 109.45818	*p* = 0.062
MCV	836.3871 ± 162.87824	924.5714 ± 61.56311	840.9672 ± 204.74586	***p* < 0.001**
MCHC	336.2120 ± 38.38356	339.9429 ± 13.24418	307.0984 ± 91.82460	***p* = 0.015**
PLT	254.2627 ± 54.93759	231.7143 ± 70.02419	261.4754 ± 58.33884	*p* = 0.144
MPV	177.3044 ± 214.35583	103.6000 ± 9.85244	372.2295 ± 316.34119	*p* = 0.057
PCT	24.8596 ± 6.29332	23.6286 ± 6.11212	18.7705 ± 9.42584	***p* < 0.001**
PDW	128.6758 ± 27.97138	123.0000 ± 20.22084	142.0984 ± 46.41936	***p* < 0.001**
RDW-CV	129.2120 ± 23.94286	132.9429 ± 11.34471	137.1475 ± 40.20482	***p* < 0.001**
MCH	294.5115 ± 28.95241	314.3714 ± 25.42010	282.9180 ± 69.45989	***p* < 0.001**
WBC	700.0046 ± 306.62644	742.1143 ± 226.21798	567.0984 ± 350.92220	*p* = 0.140
LY #	229.1106 ± 161.25184	262.7143 ± 84.71842	223.1311 ± 118.21639	***p* = 0.019**
NLR	197.8157 ± 97.44601	170.4286 ± 147.26313	191.5082 ± 149.43902	***p* = 0.003**

Because the variables did not meet the assumption of normality, the Kruskal–Wallis H test was applied for statistical comparisons. “#” denotes absolute cell counts, whereas “%” denotes relative percentages. Bold values indicate statistically significant differences (*p* < 0.05).

**Table 2:** Evaluation of Classification Performance.

		Predicted Class by the Model		
		Control Group	Alcohol Use Disorder	Substance Use Disorder
Actual Class	Control Group	T_Control_	F_Alcohol1_	F_Substance1_
	Alcohol Use Disorder	F_Control1_	T_Alcohol_	F_Substance2_
	Substance Use Disorder	F_Control2_	F_Alcohol2_	T_Substance_

Tcontrol: Correctly classified control group. Talcohol: Correctly classified alcohol use disorder group. Tsubstance: Correctly classified substance use disorder group. Falcohol1: Control group misclassified as alcohol use disorder. Fsubstance1: Control group misclassified as substance use disorder. Fcontrol1: Alcohol use disorder misclassified as control group. Fsubstance2: Alcohol use disorder misclassified as substance use disorder. Fcontrol2: Substance use disorder misclassified as control group. Falcohol2: Substance use disorder misclassified as alcohol use disorder.

**Table 3:** Definitions of Symbols Used in Performance Metric Formulas.

Accuracy	Overall Classification Performance
TPᵢ (True Positive)	Correct predictions for class i
FPᵢ (False Positive)	Incorrect predictions as class i
FNᵢ (False Negative)	Missed cases for class i
Precisionᵢ	Precision value for class i
Sensitivityᵢ (Recallᵢ)	Sensitivity (recall) value for class i

**Table 4:** Performance of Machine Learning Algorithms Across Study Groups.

Method	Accuracy (%)	Precision	Sensitivity	F1-Score	Class
Decision Trees	69.7	0.81	0.82	0.81	Control
		0.33	0.31	0.32	Alcohol
		0.66	0.66	0.66	Substance
Naive Bayes	73.7	0.84	0.80	0.82	Control
		0.39	0.60	0.47	Alcohol
		0.85	0.67	0.75	Substance
k-Nearest Neighbors (k-NNs)	75.0	0.75	0.96	0.84	Control
		0.50	0.20	0.29	Alcohol
		0.84	0.61	0.70	Substance
Logistic Regression	78.9	0.83	0.92	0.87	Control
		0.54	0.60	0.57	Alcohol
		0.88	0.61	0.72	Substance
XGBoost Classifier	78.9	0.84	0.92	0.88	Control
		0.56	0.40	0.47	Alcohol
		0.77	0.72	0.75	Substance
Artificial Neural Networks (ANNs)	79.8	0.86	0.92	0.89	Control
		0.57	0.49	0.52	Alcohol
		0.77	0.72	0.75	Substance
Support Vector Machine (SVM)	80.3	0.82	0.92	0.87	Control
		0.57	0.57	0.57	Alcohol
		0.93	0.67	0.78	Substance
Random Forest	81.6	0.80	0.98	0.88	Control
		0.75	0.43	0.55	Alcohol
		0.89	0.69	0.78	Substance

## Data Availability

The data presented in this study are available on request from the corresponding author due to ethical and privacy restrictions.
